# Supplementary material for: 1q gain bypasses the selective barrier against aneuploidy in RPE differentiation via wild-type co-culture rescue
Source: Nat Commun. 2025 Nov 25;16:11627. doi: 10.1038/s41467-025-66766-w (PMC12749988; doi:10.1038/s41467-025-66766-w)

1q gain bypasses the selective barrier against aneuploidy in RPE differentiation via wild-type co-culture rescue

Supplementary information

Supplementary Table 1. hESC lines, passage range, karyotype and label 2
Supplementary Fig. 1. Genetic characterization of all lines used in the study. 3
Supplementary Fig. 2. Immunostaining of the hESC-derived RPE. 4
Supplementary Fig. 3. Identification of the different cell populations in the scRNAseq data 5
Supplementary Table 2. Genes used to generate the scores to mark the different RPE properties 6
Supplementary Table 3. GSEA of the genes driving the differences between the RPE derived from different hESC lines 7
Supplemental Table 4. List of genes used to identify the non-RPE cell clusters in the scRNAseq 8
Supplementary Table 5. Genetic imbalances detected by scDNAseq 9
Supplementary Table 6: Lines and number replicates for the experiments shown in figure 4 11
Supplementary Fig. 4. Whole dish/well images of the differentiation experiments 12
Supplementary Fig. 5. Heatmaps of gene expression per day and cell type 13
Supplementary Table 7. Genes in Figure S5a 14
Supplementary Table 8. Taqman assays (Thermo Fisher) used in RT-qPCR 15
Supplementary Table 9. Primary and secondary antibodies 15
Supplementary Fig. 6. Gating strategy for Flow Cytometry 16
Supplementary Fig. 7. Flowchart of the steps to generate a reference set for the inferCNV analysis of scRNAseq data 16

Supplementary Table 1. hESC lines, passage range, karyotype and label

The mutations in cancer-related genes were identified by targeted resequencing and have been previously reported in Al Delbany et al[1]. Not all lines and sublines were tested, for those that were, we indicate the results for the closest passage (p) to that we used in this study. We here only indicate the *de novo* variants, including their type and allelic frequency (AF). Syn: synonymous change, Miss: missense change

hESC line	Passage	Karyotype	Breakpoints (size)	Label	De novo oncomutations
VUB01	61	46, XY			None, p66
VUB01^{17q}	78	46, XY dup(17)(q21.31qter)	dup(17): 41225000-81000000 (39.8 Mb)		Not tested
VUB01$^{1q21.1q31.1}$	83	46, XY dup(1)(q21.1q31.1)	dup(1): 143800000-187400000 (43.6 Mb)	mKate	Not tested
VUB01$^{20q11.21}$	129	46, XY dup(20)(q11.21)	dup(20): 29650000-31975000 (2.325 Mb)	GFP	None, p117
VUB02	8-12	46, XY			None, p6
VUB02^{iso20}	39	46, XY del(20)(p13-p11.21) dup(20)(q11.21qter)	del(20): 25000-25675000 (26.65 Mb) dup(20): 29575000-62775000 (33.2 Mb)	mCherry	Not tested
VUB03	24	46, XX			*KMT2C*, c.2926G>A, Miss, AF=0.05, p16
VUB03$^{1q21.1qter}$	19	46, XX dup(1)(q21.1qter)	dup(1): 144300000-247600000 (103 Mb)	Pacific Blue	Not tested
VUB03$^{1q32.1}$	34	46, XX dup(1)(q32.1)	dup(1): 202475000-205900000 (3.43 Mb)	Venus	Not tested
VUB03$^{20q11.21}$	76	46, XX dup(20)(q11.21)	dup(20): 29650000-33850000 (4.2 Mb)	Venus	Not tested
VUB04	19	46, XX			*MET*, c.4071G>C, Sun AF=0.5, p16
VUB07	24	46, XX			None, p26
VUB13^{18q}	52-58	46,XX dup(5)(q21.3qter) del(18)(q21.2qter)	dup(5): 105325000-138475000 (33.15 Mb) del(18): 48650000-77850000 (29.2 Mb)		Not tested
VUB14	20-28	46, XX			None, p20
VUB14^{18q}	49-54	46, XX dup(7)(p22.3pter) del(18q)(q21.32qter)	dup(7): 125000-31950000 (31.825 Mb) del(18): 56850000-77850000 (21 Mb)		TP53, c.404G>T, Miss, AF=1, ERCC2 c.456G>T, Syn, AF=0.5, p88
VUB14$^{20q11.21}$	35	46, XX dup(20)(q11.21)	dup(20): 29650000-31375000 (1.72 Mb)	mCherry	Not tested
VUB19^{1q}	60	46, XX dup(1)(q21.1qter)	dup(1):144300000-247600000		CSMD3, c.6952G>T, Miss, AF=0.43, POLR2A, c.2137G>A, Miss, AF=0.5, PPM1D, c.1558C>T, stop, AF=0.17, CUL4B, c.610+9G>A, Miss, AF=0.50, p60
VUB32	8	46, XX			Not tested

Supplementary Fig. 1. Genetic characterization of all lines used in the study.

The panels below show the copy-number plots obtained by whole genome shallow sequencing

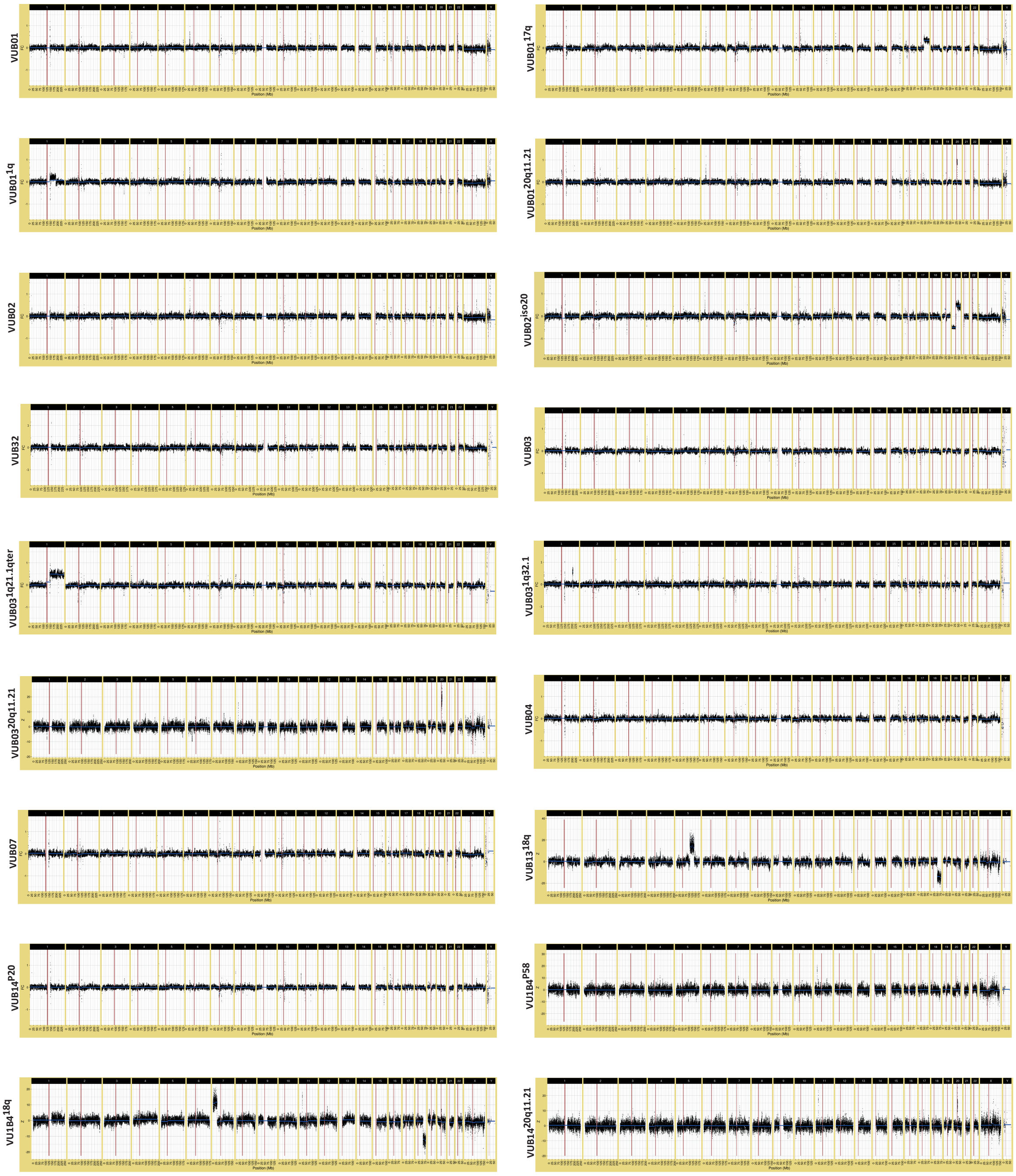

Supplementary Fig. 2. Immunostaining of the hESC-derived RPE.

The cells were stained for BEST1, PMEL, ZO-1, PAX6 and NANOG, at the end-point of differentiation.

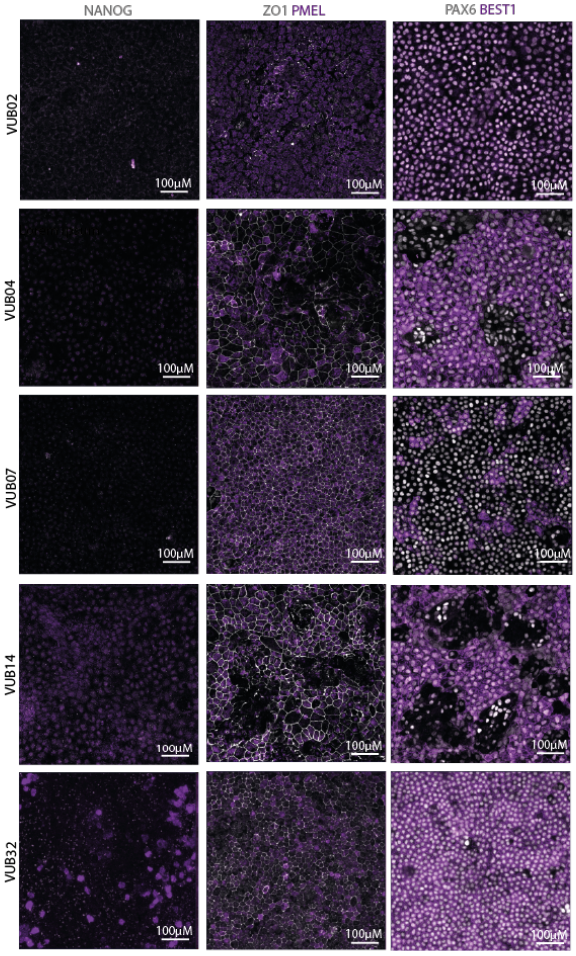

Supplementary Fig. 3. Identification of the different cell populations in the scRNAseq data

a. UMAP of the hESC-derived RPE relative to undifferentiated hESC

b. Cell cycle stage for each cell

c. Percentages of the cell cycle stages in each cluster

d. UCell scoring for each cluster for all tested cell types

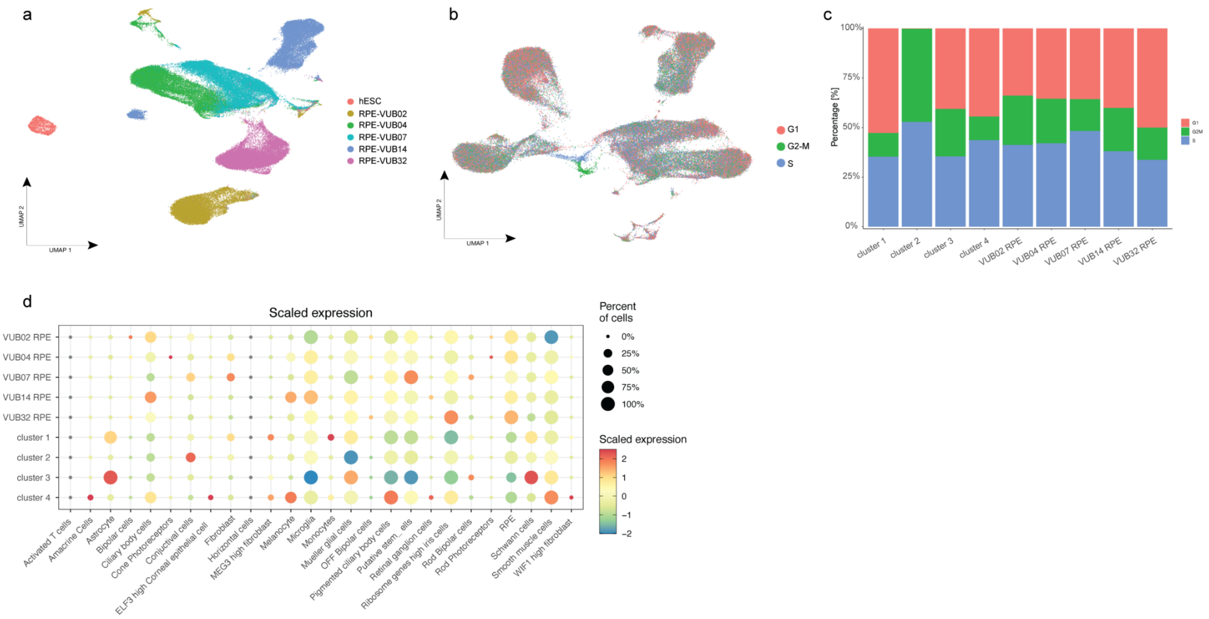

Supplementary Table 2. Genes used to generate the scores to mark the different RPE properties

Pigment synthesis	*TYR, DCT, TYRP1, SLC45A2, MITF, OCA2, GPR143, SLC24A5, KIT*
Visual cycle	*RBP2, RBP5, LRAT, ALDH1A2, RPE65, RLBP1, ABCA4, RDH11, RDH5, RBP1, CRABP1*
Phagocytic pathway	*CD81, MFGE8, CTSD, ANXA2, ITGAV, ITGB5, GAS6, CD36, MERTK, MYO7A, PROS1, TLR4, LAMP2*
Tight junction	*CLDN4, CLDN7, CRB3, MAGI2, MAGI1, OCLN, TJP3, CLDN2, CLDN3, PARD6A, MYO7A, RAB3B, CLDN5, CALM1, MPDZ, CLDN19, JAM2, PARD3, JAM3, PRKCI, CLDN12, TJAP1, MAGI3, CLDN1, CLDN11, CLDN15, F11R, PTEN, TJP2, CDC42, CDK4, CTNNA1, TJP1, ARHGEF2, RAB13, ACTB*
Ion channels	*ATP1A3, ATP1B2, ATP1B1, ATP1A1, ATP1A2, ATP1B3, SLC12A6, NEDD4L, SLC5A6, SCNN1A, SCN1B, SLC13A3, SLC23A2, SLC4A4, KCND2, KCNAB1, KCNN3, KCNC3, KCNN1, KCNAB3, KCNE3, KCNK2, KCNK15, ABCC6, ABCC5, CLCN5, CLCN4, CLCN6, CLCN3, CACNA1C, CACNB2, CACNA1G, ATP2B4*
Pluripotency	*LIN28A, POU5F1, NANOG, SOX2*

Supplementary Table 3. GSEA of the genes driving the differences between the RPE derived from different hESC lines

Cell line	GSEA ID	Gene set name	p.adjusted
VUB02	hsa03010	Ribosome	1,20E-10
VUB02	hsa05171	Coronavirus disease - COVID-19	1,31E-10
VUB02	hsa04978	Mineral absorption	1,07E-03
VUB04	hsa04978	Mineral absorption	2,72E-04
VUB04	hsa05171	Coronavirus disease - COVID-19	2,72E-04
VUB04	hsa04926	Relaxin signaling pathway	3,08E-03
VUB07	hsa03010	Ribosome	5,94E-05
VUB07	hsa05171	Coronavirus disease - COVID-19	1,13E-04
VUB07	hsa04978	Mineral absorption	1,30E-03
VUB07	hsa04210	Apoptosis	1,40E-03
VUB07	hsa04976	Bile secretion	5,20E-03
VUB14	hsa05171	Coronavirus disease - COVID-19	1,97E-09
VUB14	hsa03010	Ribosome	1,54E-08
VUB14	hsa05208	Chemical carcinogenesis - reactive oxygen species	9,18E-04
VUB32	hsa03010	Ribosome	2,21E-08
VUB32	hsa05171	Coronavirus disease - COVID-19	1,90E-07
VUB32	hsa04141	Protein processing in endoplasmic reticulum	5,17E-04
VUB32	hsa05205	Proteoglycans in cancer	5,17E-04
VUB32	hsa04978	Mineral absorption	1,89E-03
VUB32	hsa00100	Steroid biosynthesis	1,94E-03
VUB32	hsa04974	Protein digestion and absorption	1,99E-03
VUB32	hsa04510	Focal adhesion	2,49E-03
VUB32	hsa00900	Terpenoid backbone biosynthesis	3,03E-03
VUB32	hsa04151	PI3K-Akt signaling pathway	3,03E-03
VUB32	hsa05230	Central carbon metabolism in cancer	3,33E-03
VUB32	hsa04933	AGE-RAGE signaling pathway in diabetic complications	3,70E-03
VUB32	hsa05416	Viral myocarditis	4,18E-03
VUB32	hsa05165	Human papillomavirus infection	9,04E-03

Supplemental Table 4. List of genes used to identify the non-RPE cell clusters in the scRNAseq

**Cell Type**	**Marker genes**
Activated T cells	*CD69*
Amacrine cells	*TFAP2A*
Astrocyte	*GFAP, NES, FABP7*
Bipolar cells	*GRM6, PCP2*
Ciliary body cells	*AQP1*
Cone Photoreceptors	*ARR3*
Conjuctival cells	*KRT4, KRT19*
ELF3 high Corneal epithelial cell	*ELF3, KRT12*
Fibroblast	*APOD, DCN*
Horizontal cells	*ONECUT1*
MEG3 high fibroblast	*MEG3*
Melanocyte	*MLANA*
Microglia	*HEXB, CST3, CX3XR1, CTSD, CSF1R, CTSS, SPARC, TMSB4X, TMEM119, P2RY12, C1QA, C1QB*
Monocytes	*CD14, CD74*
Mueller glial cells	*CRABP1, RLBP1, GLUL, APOE, AQP4, CLU, VIM, KCNJ2, KCNJ10, S100A16, DKK3,V SX2*
OFF Bipolar cells	*GRIK1*
Pigmented ciliary body cells	*CLU, CPAMD8, PCP4, TFPI2, SGK1, HSD17B2, DCT, MLANA, COL9A1*
Putative stem cells	*MSX1, MSX2, DCT, OTX2, NTF3, SIX3, CLU, TFPI2, SGK1, WNT2B*
Retinal ganglion cells	*SNCG*
Ribosome genes high iris cells	*RPL34, RPL17*
Rod Bipolar cells	*PRKCA*
Rod Photoreceptors	*RHO*
RPE	*RPE65, BEST1, MITF*
Schwann cells	*CD9, LGI4*
Smooth muscle cells	*PCP4, ACTA2, CDH5, CALD1, CNN1, EMILIN2, HEXIM1, HRH2, MLNR, TAGLN*
WIF1 high fibroblast	*WIF1*

Supplementary Table 5. Genetic imbalances detected by scDNAseq

Line	Group	Nr of cells	Genetic imbalances
VUB02	Chaotic	1	Monosomy 1, dup(4)(q32.2q34.3), monosomy 5, dup(6)(q23.2q24.3), dup(6)(q13q14.2), del(8)(q24.11q24.3), monosomy 16
		1	del(1)(p21.2q44), monosomy 2, del(3)(p26.3p21.31), del(3)(p12.2q21.2), del(3)(q21.2q29), del(4)(p15.2q28.3), del(4)(q31.3q35.2),del(5)(q31.2q34), del(6)(q23.3q25.3), del(7)(p14.3q36.3), monosomy 8, del(9)(p24.1p11.2), del(9)(q33.3q34.3), del(10)(p13q26.13), del(11)(q23.3q24.3), del(12)(p13.33q13.13), del(15)(q21.2q26.3), del(16)(p13.13p11.2), del(18)(p11.31p11.21), del(18)(q12.1q23), del(12)(q15q24.33), del(19)(p13.2p13.11), del(20)(p13p11.23), del(19)(q13.11q13.43), del(20)(q13.13q13.33), del(22)(q11.21q13.2)
		1	dup(1)(p22.3q43), dup(2)(p16.1p13.3), dup(2)(q13q22.2),dup(2)(q35q37.3),trisomy 3, trisomy 4, trisomy 5, trisomy 6, dup(7)(q21.12q22.1), dup(8)(q12.1q24.3), dup(9)(p24.3p13.2), dup(9)(q31.1q34.11), dup(10)(p13p12.1) , dup(10)(q22.1q26.3), dup(11)(p15.5p15.1), dup(11)(q11q13.1), dup(12)(q12q24.33), dup(13)(q21.32q32.2), dup(14)(q21.2q32.11), dup(15)(q23q26.3), dup(16)(p13.3p13.13), dup(16)(q12.1q24.3), trisomy 17, dup(18)(q11.1q23), dup(19)(q11q13.41), dup(20)(q11.1q12) dup(21)(q11.2q22.3)
		1	Trisomy 1, trisomy 5, trisomy 8, trisomy 11, trisomy 12, dup(13)(q12.11q34), trisomy 16, trisomy 20, dup(21)(q11.2q22.3)
		1	Trisomy 4, trisomy 6, trisomy 7, trisomy 8, del(9)(q12q34.3), trisomy 10, trisomy 11,trisomy 12, dup(13)(q12.12q34), trisomy 13, trisomy 19 dup(21)(q11.2q22.3) dup(22)(q11.1q13.33)
	not inferCNV	1	del(1)(p36.12p34.3), del(9)(p21.3p13.3), del(1)(p22.1p11.2), del(11)(p15.1p13)
		1	del(12)(p13.33p12.3)
		1	dup(10)(q21.2q22.2)
		1	dup(4)(q28.3q34.3)
		1	del(17)(q21.31q25.3)
		1	dup(1)(q32.1q44)
		1	dup(13)(q21.32q31.3)
		1	del(13)(q14.3q31.3)
		1	dup(3)(q25.31q29), dup(5)(q32q35.3)
		1	del(6)(q13q14.1), del(10)(q21.2q22.1), del(15)(q15.2q21.3), del(21)(q11.2q22.12)
		1	del(3)(q26.33q28), del(6)(q21q27), del(18)(q21.2q22.1), del(3)(q13.12q25.1), del(1)(q43q44), del(2)(p25.3p24.2), del(3)(p24.2p22.2)
		1	del(7)(q21.11q21.3), del(7)(q31.2q34), del(15)(q21.1q21.3)
		1	del(7)(p14.3p11.2)
		2	dup(4)(q21.23q22.2)
		3	dup(7)(q21.11q21.12)
		5	dup(5)(q14.3q15)
	inferCNV	1	dup(13)(q12.11q34)
		1	Trisomy 12
		1	del(9)(p11.2q34.3)
		1	Monosomy 1, monosomy 5
		1	Trisomy 20
		1	dup(4)(q21.3q35.2)

		4	dup(9)(q21.11q34.3)
VUB04	chaotic	1	Trisomy 1, trisomy 2, trisomy 4, trisomy 5, trisomy 17, dup(21)(q11.2q22.3)
		1	Trisomy 2, trisomy 3, trisomy 5, trisomy 6, trisomy 9, trisomy 11, trisomy 12, trisomy 16, dup(13)(q11q34), trisomy 17, trisomy 18, trisomy 19, dup(21)(q11.2q22.3)
		1	Trisomy 6, trisomy 7, trisomy 18, dup(21)(q11.2q22.3)
	not inferCNV	1	del(2)(q14.1q14.3), del(2)(q22.2q23.3), del(8)(q24.11q24.22), del(9)(p24.1p13.3)
	inferCNV	1	dup(2)(q33.2q35)
		1	dup(10)(q11.23q26.3)
		1	dup(4)(q22.2q35.2)
		1	isochromosome 20q
		3	del(9)(q21.11q34.3)
		8	dup(1)(q21.3q44)
VUB07	chaotic	1	Trisomy 1, trisomy 3, trisomy 4, trisomy 5, trisomy 6, trisomy 8, trisomy 9, trisomy 11, trisomy 12, dup(13)(q11q34), trisomy 15, trisomy 19
	not inferCNV	1	dup(11)(q22.3q25)
		1	dup(7)(p22.1p15.2)
		1	dup(5)(q13.3q15), dup(11)(q14.3q22.3)
		1	del(6)(q16.3q21), dup(6)(q21q22.31), dup(6)(q23.3q24.3)
		1	del(17)(q25.1q25.3)
		1	dup(14)(q22.1q24.3), dup(20)(q12q13.13)
		3	dup(4)(q21.23q22.3)
	inferCNV	1	Monosomy 1, del(7)(p12.1q11.21) del(12)(p13.33p11.1)
		1	del(9)(q21.11q34.3)
		1	Trisomy 8
		1	Trisomy 9
		1	del(1)(p36.33q11)
		1	dup(13)(q12.11q31.1), del(13)(q31.1q34)
		5	dup(15)(q14q24.2) dup(20)(q11.21q13.2)
		5	dup(20)(q11.21q13.2)

Supplementary Table 6: Lines and number replicates for the experiments shown in figure 4

Each replicate represents an independent differentiation experiment.

hESC line	Replicates
VUB01	3
VUB02	4
$VUB03^{1q21.1qter}$	1
$VUB01^{1q}$	5
$VUB01^{17q}$	4
$VUB14^{20q11.21}$	4
$VUB03^{20q11.21}$	2
$VUB01^{20q11.21}$	4
$VUB02^{iso20q}$	4
$VUB14^{18q}$	7
$VUB13^{18q}$	6

Supplementary Fig. 4. Whole dish/well images of the differentiation experiments
Representative examples of the entire dishes/wells after RPE differentiation, prior to colony picking for all lines included in Figure 4.

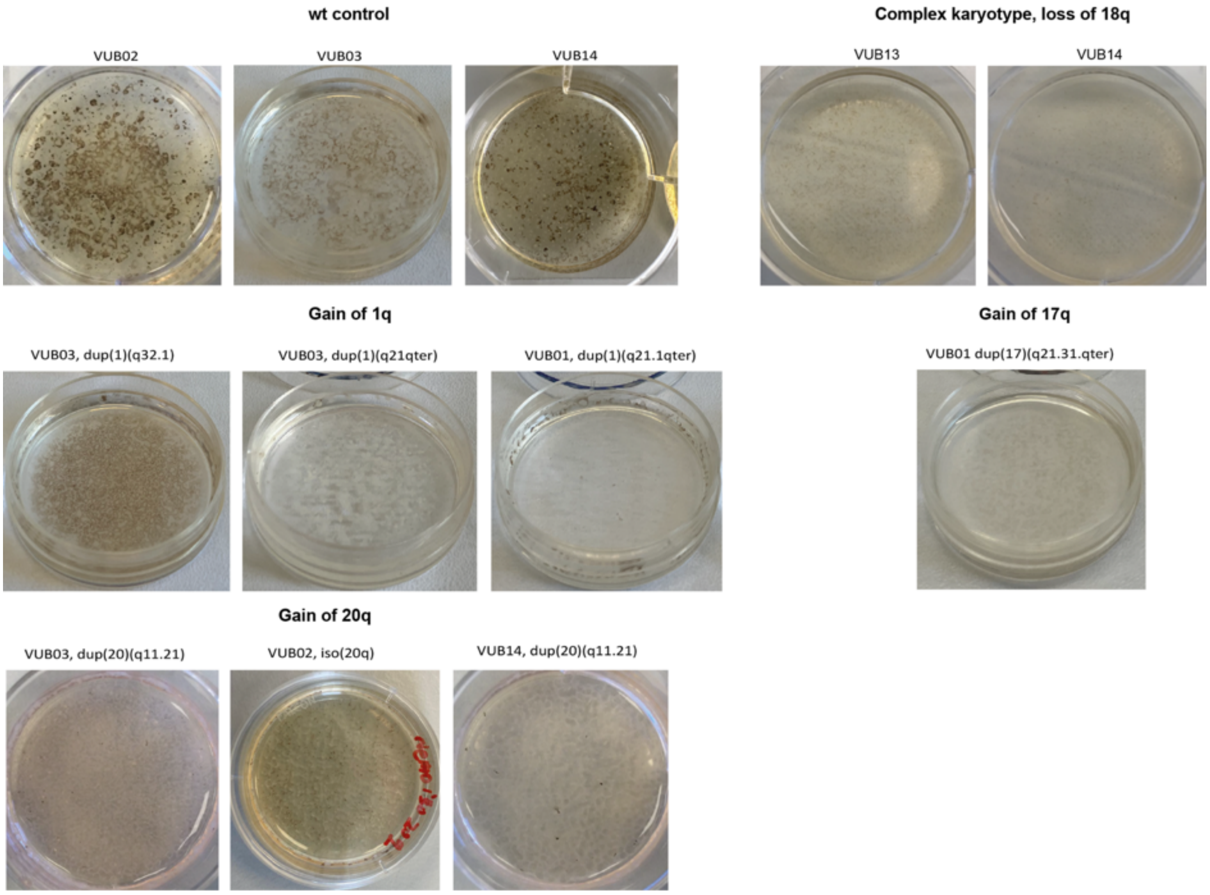

Supplementary Fig. 5. Heatmaps of gene expression per day and cell type

a. Heatmap of the expression of the top differentially expressed genes during the first two days of spontaneous differentiation, the list of genes can be found in the supplementary table 7.

b. Heatmap of the expression of cell type specific genes in the different clusters at day 7 of spontaneous differentiation

c. Heatmap of the expression of cell type specific genes in the different clusters at day 14 of spontaneous differentiation

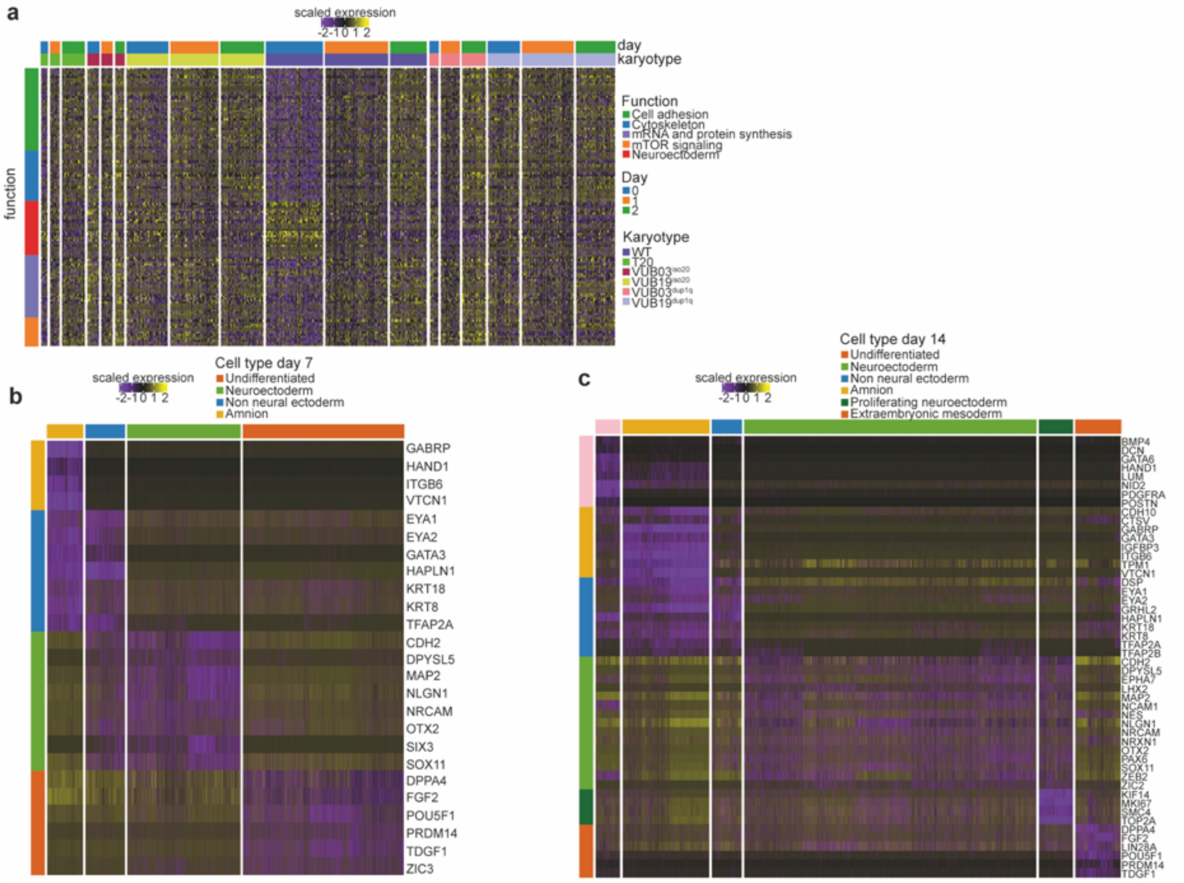

Supplementary Table 7. Genes in Figure S5a

Function	Genes
Cell adhesion	*ACTN1, ADAM9, AHNAK, ANXA2, CALD1, CARD11, CASP3, CDH13, CDH2, CLIC1, COL5A2, CTGF, CYR61, DSP, ENO1, EPCAM, EPS15, FBLIM1, FOXO3, ITGAV, ITGB1, ITGB5, LDHA, LIMA1, LIMS1, LPP, MYL12A, NTNG1, OCLN, PERP, PFN1, PKP2, PRICKLE1, RAPGEF1, SLC3A2, SLC7A1, SNX9, TENM2, TLN1, VCL, ZYX*
Cytoskeleton	*ACTR3, ARHGAP10, CAMK2D, CAP2, CAPNS1, CDC42BPA, CLIP1, COL1A1, COTL1, FLNC, FRMD6, KIF1A, MAP1B, MID1, MYL12B, SORBS2, SVIL, TAGLN, TMSB4X, TTN, TUBA1A, TUBB2B, TUBB6, VASP, VIM*
mRNA and protein synthesis	*BNC2, ECPAS, FAU, GARS, HECW1, HINT1, HIST1H1D, HIST1H1E, KHDRBS2, MBNL1, MGAT4C, NARS, NME2, POMP, PSMA7, PSMC1, PSMC3, RPL22L1, RPL27, RPL35, RPL38, RPLP1, SAMD4A, SARS, SNRPB2, SYF2, UBC, USP53, WARS, ZNF577*
mTOR signaling	*CYP51A1, EIF2S2, GBE1, HMGCR, HMGCS1, IDI1, ME1, MTHFD2, PSAT1, SLC2A1, SLC7A11, SLC7A5, SQLE, SQSTM1*
Neuroectoderm	*ACTB, ADGRL1, AGRN, AP2A1, AP2M1, ASTN2, AUTS2, BRINP1, CACNA1H, CAMK1D, CDKL5, CFL1, CMTM8, COL4A1, COL6A1, COL6A2, CTNNA2, CTNND2, DAB1, DAB2IP, DLGAP2, EIF4A3, EPHA6, EVL, FGFR1, FGFR2, GPRC5B, HLTF, HSPG2, KCNQ2, KLF7, LAMA2, LDB1, MSI2, MYH9, NAV1, NES, NRP2, NRXN1, OPCML, PAK3, PBX1, PDLIM7, PGAP1, PIP5K1C, PLCG1, PLPP3, PLXNA1, PLXNB1, PRKCZ, PRKD1, PRKG1, PSMD6, PTCH1, RGMA, ROBO2, RORA, RPL18, RPL3, RPL7, RPL8, SEMA4D, SFRP1, SLIT2, SORL1, SPTBN2, TBCD, TENM4, TUBB3, TUBB4B, UNC5B, UNC5D, VLDLR, WNK2*

Supplementary Table 8. Taqman assays (Thermo Fisher) used in RT-qPCR

Gene	Reference
BEST1	Hs00188249_m1
NANOG	Hs02387400_g1
PAX6	Hs00240871_m1
PMEL	Hs00173854_m1
POU5F1	Hs00742896_s1
RPE65	Hs00165642_m1
GUSB	4326320E
ID1 – copy number	Hs01892845_cn
RNASEP – copy number	4403326

Supplementary Table 9. Primary and secondary antibodies

**Primary antibodies**	**Reference**
BEST1 (1:200)	Merck Millipore, MAB5466
GATA4 (1:400)	Cell Signaling Technology, 36966S
HNF4α (1:200)	Santa Cruz Biotechnology, sc-374229
NANOG (1:200)	Cell signaling Technology, 4903
OCT3/4 (1:200)	Santa Cruz Biotechnology, sc-5279
OCT4A (1:400)	Cell Signaling Technology, 2840
PAX6 (1:200)	Invitrogen, MA5-32409
PAX6 (1:500)	Biolegend, 901301
PAX6 (1:200)	Abcam, AB78545
PMEL (1:200)	Invitrogen, MA1-34759
ZO-1 (1:200)	Invitrogen, 61-7300
**Secondary antibodies**	
Alexa Fluor 488 goat anti-mouse IgG (H+L) (1:200)	Thermo Fisher, A-11001
Alexa Fluor 594 donkey anti-rabbit IgG (H+L) (1:200)	Thermo Fisher, A-21207
Alexa Fluor 647 donkey anti-mouse IgG (H+L) (1:200)	Thermo Fisher, A-31571
**Conjugated antibody**	
Hoechst 33342, trihydrochloride, trihydrate (1:2000)	Invitrogen, H3570

Supplementary Fig. 6. Gating strategy for Flow Cytometry

Representative images on the gating strategy used for the flow cytometry of fluorescently labelled cells

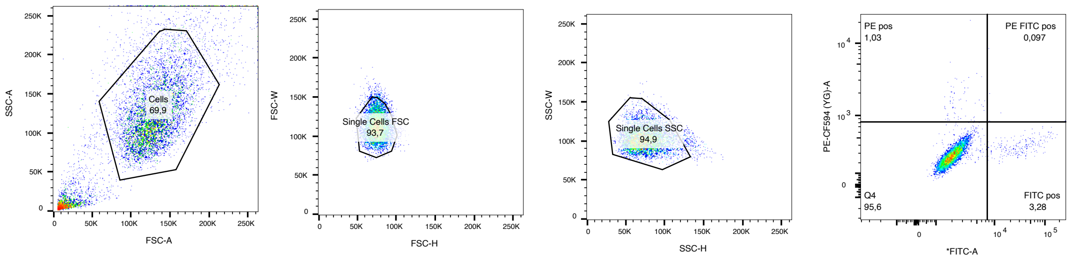

Supplementary Fig. 7. Flowchart of the steps to generate a reference set for the inferCNV analysis of scRNAseq data

Steps in the process to select genetically balanced cells to establish a reference set during inferCNV analysis

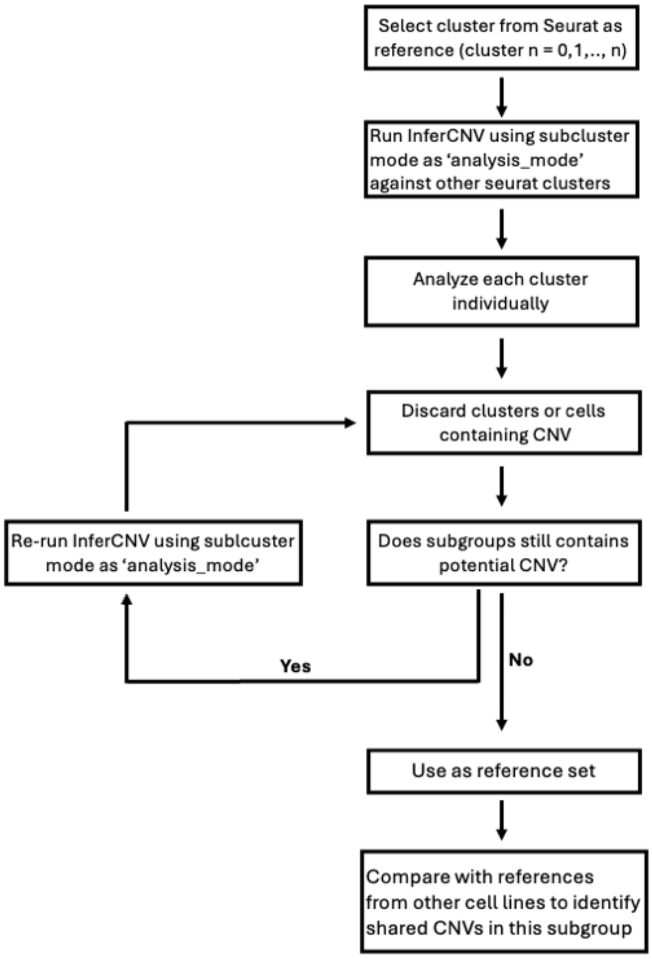

Supplement: Supplementary file 1 — Supplementary Information [file 41467_2025_66766_MOESM1_ESM.pdf]
