# Supplementary material for: 1q gain bypasses the selective barrier against aneuploidy in RPE differentiation via wild-type co-culture rescue
Source: Nat Commun. 2025 Nov 25;16:11627. doi: 10.1038/s41467-025-66766-w (PMC12749988; doi:10.1038/s41467-025-66766-w)
Supplement: Supplementary file 2 — Description of Additional Supplementary Files [file 41467_2025_66766_MOESM2_ESM.pdf]

**Description of Additional Supplementary Files**

File Name: Supplementary Data 1
Description: Complete results for GSEA using the H library, on the bulk RNA sequencing of $RPE^{1q}$ vs $RPE^{wt}$

File Name: Supplementary Data 2
Description: Complete results for GSEA using the C2 library, on the bulk RNA sequencing of $RPE^{1q}$ vs $RPE^{wt}$

File Name: Supplementary Movie 1

Description: Fragment of a live cell imaging for 24 hours to visualize DNA and microtubules, it shows hPSC undergoing tripolar spindle mitotic divisions.
