# Supplementary material for: 1q gain bypasses the selective barrier against aneuploidy in RPE differentiation via wild-type co-culture rescue
Source: Nat Commun. 2025 Nov 25;16:11627. doi: 10.1038/s41467-025-66766-w (PMC12749988; doi:10.1038/s41467-025-66766-w)
Supplement: Supplementary file 6 — Reporting Summary [file 41467_2025_66766_MOESM6_ESM.pdf]

nature portfolio

Corresponding author(s): Claudia.Spits@vub.be

Last updated by author(s): 22/10/2025

Reporting Summary

Nature Portfolio wishes to improve the reproducibility of the work that we publish. This form provides structure for consistency and transparency in reporting. For further information on Nature Portfolio policies, see our Editorial Policies and the Editorial Policy Checklist.

Statistics

For all statistical analyses, confirm that the following items are present in the figure legend, table legend, main text, or Methods section.

n/a	Confirmed	
☐	☒	The exact sample size (*n*) for each experimental group/condition, given as a discrete number and unit of measurement
☐	☒	A statement on whether measurements were taken from distinct samples or whether the same sample was measured repeatedly
☐	☒	The statistical test(s) used AND whether they are one- or two-sided<br>*Only common tests should be described solely by name; describe more complex techniques in the Methods section.*
☒	☐	A description of all covariates tested
☒	☐	A description of any assumptions or corrections, such as tests of normality and adjustment for multiple comparisons
☒	☐	A full description of the statistical parameters including central tendency (e.g. means) or other basic estimates (e.g. regression coefficient) AND variation (e.g. standard deviation) or associated estimates of uncertainty (e.g. confidence intervals)
☐	☒	For null hypothesis testing, the test statistic (e.g. *F*, *t*, *r*) with confidence intervals, effect sizes, degrees of freedom and *P* value noted<br>*Give P values as exact values whenever suitable.*
☒	☐	For Bayesian analysis, information on the choice of priors and Markov chain Monte Carlo settings
☒	☐	For hierarchical and complex designs, identification of the appropriate level for tests and full reporting of outcomes
☒	☐	Estimates of effect sizes (e.g. Cohen's *d*, Pearson's *r*), indicating how they were calculated

Our web collection on statistics for biologists contains articles on many of the points above.

Software and code

Policy information about availability of computer code

Data collection	No software was used.
Data analysis	The software programs used in this study were Applied Biosystems Copy Caller v.2.1, Cellranger-dna mkfastq version 1.1.0, R package Aneufinder version 3.14, R package copynumber version 3.15, CellRanger 3.1.0, Seurat R package version 4.3, Scenic and Liana.

For manuscripts utilizing custom algorithms or software that are central to the research but not yet described in published literature, software must be made available to editors and reviewers. We strongly encourage code deposition in a community repository (e.g. GitHub). See the Nature Portfolio guidelines for submitting code & software for further information.

Data

Policy information about availability of data

All manuscripts must include a data availability statement. This statement should provide the following information, where applicable:

- Accession codes, unique identifiers, or web links for publicly available datasets
- A description of any restrictions on data availability
- For clinical datasets or third party data, please ensure that the statement adheres to our policy

Details on derivation and results of characterization, including tests for pluripotency, of all hESC lines used in this study can be found at the Open Science Framework repository [https://osf.io/esmz8/]. The lines are registered in the EU hPSC registry [https://hpscreg.eu/], and available upon request.
The RNA sequencing count tables and all the data supporting the figures in this paper can be found at the Open Science Framework repository [https://osf.io/y8tzh/] (https://doi.org/10.17605/OSF.IO/Y8TZH).

Raw sequencing data of human samples is considered personal data by the General Data Protection Regulation of the European Union (Regulation (EU) 2016/679), because SNPs can be extracted from the reads, and cannot be publicly shared. The data can be obtained from the corresponding author upon reasonable request and after signing a Data Use Agreement.

Research involving human participants, their data, or biological material

Policy information about studies with human participants or human data. See also policy information about sex, gender (identity/presentation), and sexual orientation and race, ethnicity and racism.

Reporting on sex and gender	Although the collection of sex-specific data was not an objective of the study, sex chromosome information was reported as part of the karyotyping analyses of the various stem cell lines, as presented in Supplementary Table 1.
Reporting on race, ethnicity, or other socially relevant groupings	Not applicable.
Population characteristics	Not applicable.
Recruitment	Not applicable.
Ethics oversight	Design and conduct complied with all relevant regulations regarding the use of human materials, and all were approved by the local ethical committee of the University Hospital UZ Brussel and the Vrije Universiteit Brussel (File number: B.U.N. 1432021000669).

Note that full information on the approval of the study protocol must also be provided in the manuscript.

Field-specific reporting

Please select the one below that is the best fit for your research. If you are not sure, read the appropriate sections before making your selection.

[x] Life sciences [ ] Behavioural & social sciences [ ] Ecological, evolutionary & environmental sciences

For a reference copy of the document with all sections, see nature.com/documents/nr-reporting-summary-flat.pdf

Life sciences study design

All studies must disclose on these points even when the disclosure is negative.

Sample size	No sample size was calculated. We aimed at including at least three biological replicates for all experiments, and choose the hESC lines based on the availability in our laboratory and their karyotypes.
Data exclusions	No data was excluded
Replication	The experiments were replicated as indicated in the manuscript, including experiments on at least three different cell lines. All outcomes are reported, and results were consistent accros replicates
Randomization	There was no randomization in this study, this was not relevant to our study, as we were testing the impact of different chromosomal abnormalities on the differentiation trajectories of stem cells
Blinding	There was no blinding in this study

Reporting for specific materials, systems and methods

We require information from authors about some types of materials, experimental systems and methods used in many studies. Here, indicate whether each material, system or method listed is relevant to your study. If you are not sure if a list item applies to your research, read the appropriate section before selecting a response.

Materials & experimental systems

n/a	Involved in the study	
[ ]	[x]	Antibodies
[ ]	[x]	Eukaryotic cell lines
[x]	[ ]	Palaeontology and archaeology
[x]	[ ]	Animals and other organisms
[x]	[ ]	Clinical data
[x]	[ ]	Dual use research of concern
[x]	[ ]	Plants

Methods

n/a	Involved in the study	
[x]	[ ]	ChIP-seq
[ ]	[x]	Flow cytometry
[x]	[ ]	MRI-based neuroimaging

Antibodies

Antibodies used

Primary antibodies: BEST1 (Merck Millipore, MAB5466); GATA4 (Cell Signaling Technology, 36966S, D3A3M), HNF4& (Santa Cruz Biotechnology, sc-374229, H-1); NANOG (Cell Signaling Technology, 4903, D73G4); OCT3/4 (Santa Cruz Biotechnology, sc-5279, C-10); OCT4A (Cell Signaling Technology, 2840, C30A3); PAX6 (Invitrogen, MA5-32409, SD08-31); PAX6 (Biolegend, 901301, Poly19013); PAX6 (Abcam, AB78545, AD2.38); PMEL (Invitrogen, MA1-34759, HMB45) and ZO-1 (Invitrogen, 61-7300).

Secondary antibodies: AF488 goat anti-mouse IgG (H+L) (ThermoFisher, A-11001); AF 594 donkey anti-rabbit IgG (H+L) (TharmoFisher, A-21207) and AF647 donkey anti-mouse IgG (H+L) (ThermoFisher, A-31571).

Conjugated antibody: Hoechst 33342, trihydrochloride, trihydrate (Invitrogen, H3570).

Validation

BEST1 (Merck Millipore, MAB5466): Western blot: 5 µg/mL of this antibody detected BEST1 in bovine retina cell lysate. Immunohistochemistry (Paraffin) Analysis: A 1:20 dilution from a representative lot detected BEST1 in mouse retina tissue sections This antibody was detected in iPSCs-derived RPE in two peer reviewed papers cited on manufacture website.
GATA4 (Cell Signaling Technology, 36966S, D3A3M): This antibody (Cell Signaling Technology, D3A3M, #36966) was validated by the manufacturer for immunofluorescence, showing positive nuclear staining in Huh7 cells and no staining in HeLa cells (negative control). Western blot analysis detected a band at the expected molecular weight (~54 kDa) in Huh7, HepG2 cell lysates, with no signal in HeLa cells. In the literature, this antibody has also been reported to detect GATA4 during endoderm differentiation of hESCs (day 3) and in neonatal rat ventricular myocytes.
HNF4α (Santa Cruz Biotechnology, sc-374229, H-1): This antibody was validated on Hep G2, Caco-2, WiDr and F9 cell lysate for Western blot (55kDa band) and on Hep G2 immunofluorescence staining.
NANOG (Cell Signaling Technology, 4903, D73G4):This rabbit mAb was validated by Western blot on NCCIT, NTERA-2 and iPS cells, by Immunohistochemical on paraffin-embedded human seminoma, and by immunofluorescent analysis on NTERA-2 cells and HeLa cells. Over 500 publications further validated this antibody.
OCT3/4 (Santa Cruz Biotechnology, sc-5279, C-10): This mouse monoclonal antibody was validated by manufacturer by Western Blot on F9, ES-D3 and NTERA-2 cell lysates, by Immunofluorescence staining of paraformaldehyde fixed multicell mouse embryo. There are 2693 publications that utilised this antibody, supporting its specificity.
OCT4A (Cell Signaling Technology, 2840, C30A3): This rabbit monoclonal antibody is validated by the manufacturer for Western blotting, immunofluorescence, and flow cytometry, with 245 citations listed on the CST website. Validation data include Western blot detection of OCT4A in NTERA2 and mouse embryonic stem cells, immunofluorescence in NTERA2 and mESCs grown on MEF feeders, and flow cytometry in Daudi and NCCIT cells, confirming specificity and expected expression patterns.
PAX6 (Invitrogen, MA5-32409, SD08-31): Validation by the manufacturer includes Western blot and immunohistochemistry on human, mouse, and rat tissues and cell lines with differential expression consistent with published PAX6 biology. Specificity was confirmed by CRISPR-Cas9 knockout in U-87 MG cells, where loss of PAX6 signal was observed by Western blot. Additional validation includes relative expression analysis across multiple human and murine cell lines, supporting antibody specificity.
PAX6 (BioLegend, 901301, clone polyclonal):Each lot of this antibody is quality control tested by the manufacturer for Western blot and immunohistochemistry on formalin-fixed, paraffin-embedded mouse brain tissue. Validation data include Western blot of human (293T) and mouse brain lysates as well as IHC staining of mouse brain, consistent with published PAX6 expression. Over 300 publications have employed this antibody, supporting its established specificity.
PAX6 (Abcam, ab78545, AD2.38): This mouse monoclonal antibody is validated for use in IHC (FFPE and frozen), immunocytochemistry, and immunofluorescence across human, mouse, and rat samples, with >50 citations in the literature. Validation data include IHC of mouse embryonic brain, rat retina, and human pancreas tissue, as well as staining of primary mouse hippocampal neurons. The observed expression is consistent with known PAX6 distribution in neuroectodermal and ocular tissues.
PMEL (Invitrogen, MA1-34759, HMB45): This antibody is validated for Western blot, immunohistochemistry, and immunofluorescence. Validation includes differential expression analysis in SK-MEL-5 cells compared to A-375 and SK-BR-3 cells, consistent with known PMEL biology. Tissue analysis confirmed expression in mouse skin and malignant human melanoma, but not in negative control tissues such as heart. Published data (37 references) and the known specificity of HMB45 for melanocytic lineage confirm antibody reliability.
ZO-1 (Invitrogen, 61-7300): This polyclonal antibody is validated for Western blot, immunoprecipitation, immunofluorescence, ELISA, and immunohistochemistry, with >900 citations. Manufacturer’s validation demonstrates expected junctional staining in epithelial cell lines (Caco-2, MDCK) but not in low-expressing Jurkat or Reh cells. Specificity has been further confirmed by decreased expression following IFNγ treatment in Caco-2 cells. Immunofluorescence in epithelial cell monolayers and immunohistochemistry of small intestine tissue confirm the expected localization of ZO-1 at tight junctions.

Eukaryotic cell lines

Policy information about cell lines and Sex and Gender in Research

Cell line source(s)

All hESC lines in this study were derived in-house in the past from spare human IVF/ICSI blastocyst stage embryos whose morphology did not fulfill the requirements for transfer or freezing. All of the patients donating embryos to derive these lines gave written consent. The lines are registered in the EU hPSC registry (https://hpscreg.eu/).

293T cell line is a gift from the Virus Production Unit of the Vrije Universiteit Brussel.

Authentication

Prior to the start of this study, cell working banks were created for each of the hESC lines, which were karyotyped by shallow whole-genome sequencing. The cells were authenticated by fingerprinting using Devyser Complete v2 kit (Devyser, Hägersten, Sweden) on ABI Genetic Analyzer + GeneMapper V6 software (Applied Biosystems)

Mycoplasma contamination	Prior to the start of this study, cell working banks were created for each of the lines, which were controlled for mycoplasma infection by PCR and were confirmed to be negative, the protocol can be found at https://osf.io/jcen3/.
Commonly misidentified lines (See ICLAC register)	Not applicable.

Plants

Seed stocks	Not applicable.
Novel plant genotypes	Not applicable.
Authentication	Not applicable.

Flow Cytometry

Plots

Confirm that:

- [ ] The axis labels state the marker and fluorochrome used (e.g. CD4-FITC).
- [ ] The axis scales are clearly visible. Include numbers along axes only for bottom left plot of group (a 'group' is an analysis of identical markers).
- [ ] All plots are contour plots with outliers or pseudocolor plots.
- [x] A numerical value for number of cells or percentage (with statistics) is provided.

Methodology

Sample preparation	Fluorescently labelled hESC with a gain of 1q and their isogenic genetically balanced counterparts (VUB031q21.1qter-BLUE and VUB031q32.1q31.1-VENUS) were co-cultured for 12 weeks. Pigmented colonies were picked (=mechanically cut out using a sharpened glass pipette) at 12 weeks of differentiation, expanded and passaged. Cells were collected at start of differentiation and two passages after colony picking by using accutase.
Instrument	BD FACSMelody flow cytometer (BD Biosciences)
Software	FlowJo (BD Biosciences)
Cell population abundance	The range was 0.2% to 25%
Gating strategy	Cells were sorted under BSL-2 conditions using a 3 laser BD FACSMelody flow cytometer (BD Biosciences). Unlabeled cells served as a negative control to establish baseline fluorescence levels for gating to sort out BLUE or VENUS expressing cells. Sorting was performed based solely on BLUE/VENUS fluorescence, with singlets identified using FSC-W and SSC-W parameters.

- [x] Tick this box to confirm that a figure exemplifying the gating strategy is provided in the Supplementary Information.
